# Insights into the cumulative effect of C*olletotrichum gloeosporioides* and* Fusarium acutatum* causing anthracnose-twister disease complex of onion

**DOI:** 10.1038/s41598-024-59822-w

**Published:** 2024-04-23

**Authors:** Ram Dutta, K. Jayalakshmi, Satish Kumar, A. Radhakrishna, Dalasanuru Chandregowda Manjunathagowda, M. N. Sharath, Vishal S. Gurav, Vijay Mahajan

**Affiliations:** 1 ICAR-Directorate of Onion and Garlic Research, Pune, Maharashtra India; 2 ICAR-Indian Institute of Horticultural Research, Hesaraghatta Lake Post, Bengaluru, Karnataka India

**Keywords:** Microbiology, Plant sciences

## Abstract

*Colletotrichum* is an important plant pathogenic fungi that causes anthracnose/-twister disease in onion. This disease was prevalent in the monsoon season from August to November months and the symptoms were observed in most of the fields. This study aimed to investigate the pathogenicity and cumulative effect, if any of *Colletotrichum gloeosporioides* and *Fusarium acutatum*. The pot experiment was laid out to identify the cause responsible for inciting anthracnose-twister disease, whether the *Colletotrichum* or *Fusarium* or both, or the interaction of pathogens and GA3. The results of the pathogenicity test confirmed that *C. gloeosporioides* and *F. acutatum* are both pathogenic. *C. gloeosporioides* caused twisting symptoms independently, while *F.acutatum* independently caused only neck elongation. The independent application of GA3 did not produce any symptoms, however, increased the plant height. The combined treatment of *C. gloeosporioides* and *F. acutatum* caused twisting, which enhanced upon interaction with GA3 application giving synergistic effect. The acervuli were found in lesions infected with *C. gloeosporioides* after 8 days of inoculation on the neck and leaf blades. Symptoms were not observed in untreated control plants. Koch's postulates were confirmed by reisolating the same pathogens from the infected plants.

## Introduction

Onion (*Allium cepa* L.), a fresh spice-vegetable, is a key component in almost every cuisine throughout the world and is also known for bioactive substances like organosulfur, flavonols, polysaccharides, saponins, and phenolic compounds^1^. Averaged over the quinquennial period of 2017 to 2021, India ranked 1st for the area (1.37 million ha) and production (24.25 million t) of onion in the world. Onion productivity (16.40 t/ha) of India ranked at 90th position being far lower than many other countries^2^. In India, onions are grown three times in a year viz. early and late *Kharif* seasons 20% each from July to October and December to January, respectively, and the rest in the *Rabi* season i.e. 60% (from December to April). Although *Kharif* onions make up only a small portion of the nation’s overall production (up to 20%), they have a big impact on price stability as they ensure a steady supply in the market during October, November, December, and January, when stored onions from the *Rabi* are not readily available^3–7^.

The anthracnose-twister for which this study was undertaken is very important during *Kharif* (rainy) season and onions are highly susceptible to the anthracnose-twister, which is currently the most devastating disease causing estimated yield losses of onion to the tune of 80–100%, depending on its severity and the growth stage of the crop^8–12^. It is a major factor responsible for low production and productivity. Onion anthracnose-twister (also known as ‘severe curl’ disease) is reported to be widespread across the world but more prevalent in the tropics and subtropics during rainy seasons. The production potential of the onion is also known to be hampered by several biotic (e.g. Purple blotch, *Stemphylium* blight, *Fusarium* basal rot, thrips, etc.) and abiotic causes (excessive rainfall, drought, salinity, temperature stress).

Effective management of the onion anthracnose-twister is still lacking and a sound management approach requires a good knowledge of the host–pathogen relationship, especially the stages at which infection and colonization occurs. The progression of anthracnose-twister disease is typically characterized by the development of characteristic lesions along with twisting of the leaves; however, the development of lesions is not always accompanied with leaf twisting.

*Colletotrichum gloeosporioides* is the causal organism of the anthracnose and twisting symptom^13–15^. The disease was first reported near Zaria, north Nigeria, in 1969 caused by *Glomerella cingulata* (the perfect state of *C. gloeosporioides*) during *kharif* (rainy) season. Panday et al.^16^ studied the polycyclic nature of *C. gloeosporioides* by initial and post penetration characterization of pathogen and reported 96-h requirement for completion of pathogen cycle and to develop typical anthracnose symptoms in onion. Another pathogen *Gibberella moniliformis* was reported to produce overlapping symptoms of twisting and abnormal neck elongation in onions due to the excessive accumulation of gibberellins^17^. Lestiyani et al.^18^ reported onion twister is caused by *Fusarium oxysporum, F. solani,* and *F. acutatum*. Though, each species playing different role in symptom development i.e., *F. solani* and *F. acutatum* causes wilting; *F. solani, F. acutatum* or *F. oxysporum* causes bulb rotting, the twisting symptoms were caused by *F. solani* or *F. acutatum*. In another report, onion seedlings inoculated with *C. gloeosporioides, F. oxysporum*, and *Meloidogyne* spp. also caused twister disease symptoms^19^.

Having gone through the existing literature, it is observed that the current state of research findings of onion-twister disease is quite confusing. The main confusion lies in ‘whether the onion twister disease is caused by *Colletotrichum* spp. alone’ or by the ‘complex of *Colletotrichum* spp. and *Fusarium* spp.’ Reports are also available which suggest the role of GA3 in onion neck elongation and twisting. Further, instances have been observed where the twisting of onion leaves occurs with no development of leaf lesions, which are otherwise often developed with the *Colletotrichum* infection. The role of GA3 that causes cell elongation is also not yet clearly understood. The already published reports have explained the role of *Fusarium* spp*.* as an underlying pathogen for onion anthracnose-twister disease. Hence, the experiments were conducted to examine and evaluate the individual and combinational roles of *C. gloeosporioides, F. acutatum,* and GA3 in causing the onion anthracnose-twister disease. Our study reveals the less important role of *Fusarium* and GA3 in the development of the anthracnose-twister disease and the disease is primarily caused by *C. gloeosporioides.* However, the concurrent presence of *Fusarium* increased the disease severity, which further increased with the presence of GA3. The information on the host–pathogen relationship and symptom development will provide further understanding of onion-twister disease and the pertinent management practices.

## Results

### Disease symptoms and associated pathogens

This study was aimed to investigate the pathogenicity and cumulative effect of *Colletotrichum gloeosporioides* and *Fusarium acutatum* known to be causing anthracnose-twister disease in onion. The pot experiment was laid out to identify the actual cause responsible for inciting anthracnose-twister disease, whether the *Colletotrichum* or *Fusarium* or both, or the interaction of pathogens and GA3. In the early stages of infection, slight neck elongation was observed, and thereafter twisting of the leaf and neck was noticed. As the infection progressed, slight curling and twisting of the leaves along with white depressed spots/lesions were observed on the leaves and neck. In later stages of infection, the neck became fully elongated, with complete rolling of leaves and plants turning chlorotic, slender and dropping ultimately. In advanced stages, the lesions turned brown and appeared as concentric rings containing orange or salmon-colored fruiting bodies. As the anthracnose and twisting became severe leading to the formation of a disease complex resulting in the neck and leaves of plants being severely affected, which ultimately led to the drying of leaves from tip giving dieback symptoms, and finally wilting or death of the plant.

To identify the fungal pathogen/s associated with onion samples showing typical twister disease, standard isolation procedures were employed as described in methods. Leaf, neck and roots showing typical anthracnose-twister symptoms were collected from onion field. Infected leaf samples were used for isolation of the pathogens, and incubated at 25 ± 2 °C. The results of isolation after 7 days of incubation at 25 ± 2 °C showed that two pathogenic fungus were associated with this disease in onion plant and were later identified as *Colletotrichum* spp. (OGRDCG1) and *Fusarium* spp. (OGRDFW1) based on their morphological characters^20–22^.

**Figure 1:**
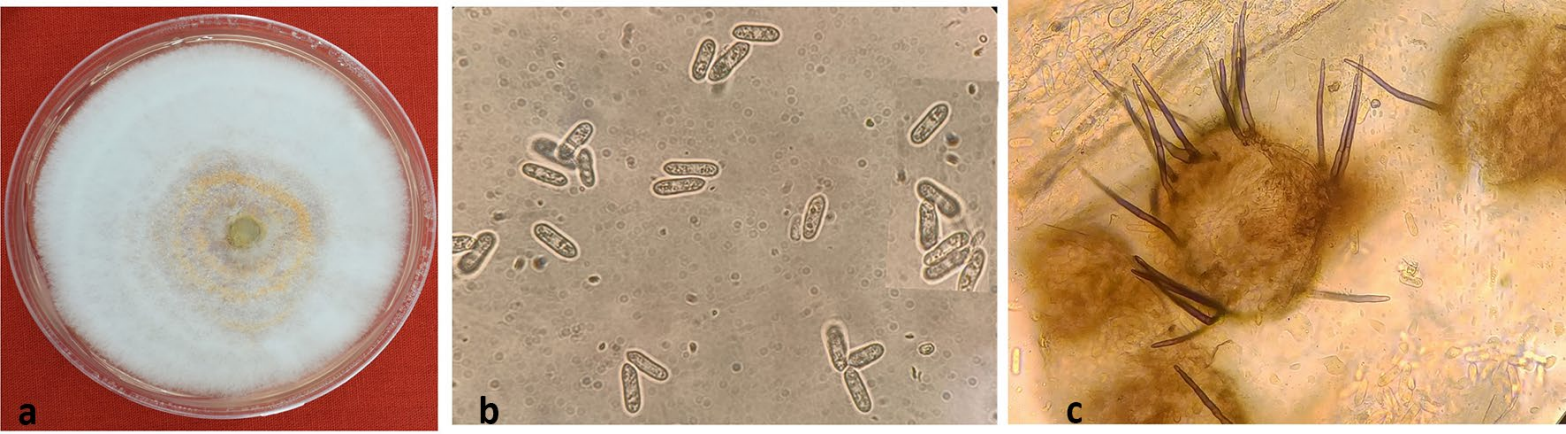
Colony morphology of *Colletotrichum gloeosporioides* (**a**) White mycelium with orange conidial mass (**b**) Conidia (**c**) Acervulli.

**Figure 2:**
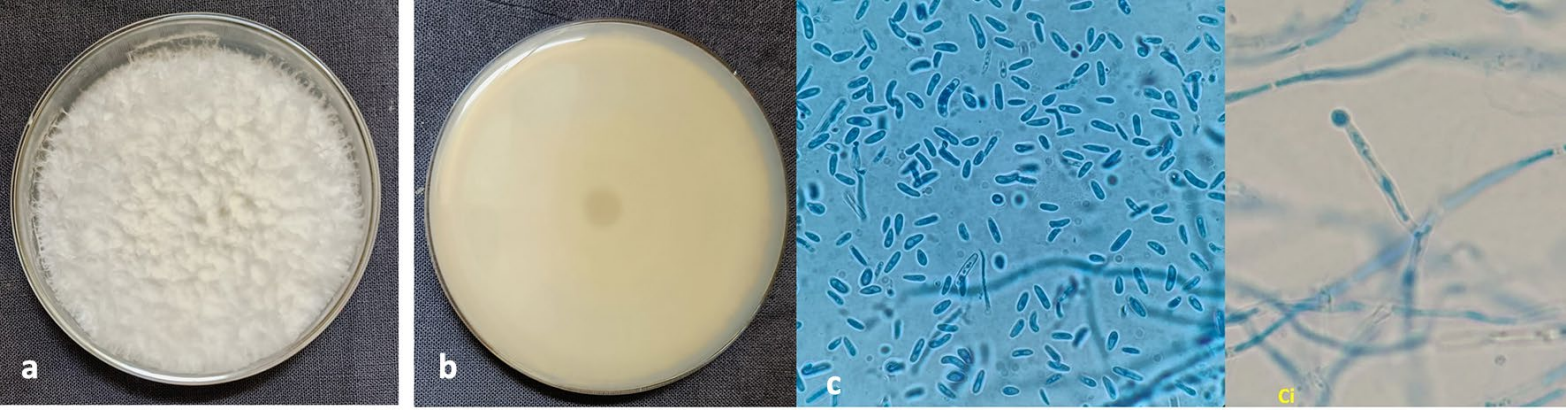
Culture morphology of *Fusarium acutatum* (**a**) Top view showing abundant white aerial mycelia, (**b**) Bottom view showing dull white coloration (**c**) Macro and microconidia (1000x) ci) Developing microconidia (In situ).

The color of *Colletotrichum* colony was white to grey with yellowish orange pigments (Fig. 1). The mycelium was septate, hyaline, conidia were single celled, hyaline cylindrical/dumbel with rounded ends having oil globules measuring 3.6–5.4 × 10.5–15.3 µm (n = 50). Acervullus with orange conidial masses confirmed the *Colletotrichum* spp. The colony of *Fusarium* showed white color on both sides (Fig. 2). When the cultures grew old, they became thick due to the growth of white aerial hyphae mat on the colony surface. The mycelium was septate, and the macroconidia were slightly curved or with bend apical cells measuring 17.8 × 3.1 μm, (n = 50) with one to two septa. Whereas, microconidia were ovoid, aseptate, measuring 4.3 × 2.1 μm, (n = 50) and chlamydospores were round, intercalary, hyaline and single. This confirmed the *Fusarium* spp. These cultures were stored at 4 °C for further studies.

### Molecular characterization of *Colletotrichum* and *Fusarium* spp.

The isolated pathogens morphologically identified as *Colletotrichum* spp. and *Fusarium* spp. were further subjected to molecular characterization through universal ITS and *tef* gene based primers. The PCR analysis for *C. gloeosporioides* and *F. acutatum* with ITS 1/4 resulted in amplicons of 487 bp and 338 bp while the amplicon size of *tef* gene was 456 and 613 bp, respectively. Upon analysis using NCBI (National Centre for Bioinformatics) BLAST tool both the isolates of OGRDCG1 and OGRDFW1 showed > 98% similarity identity with *Colletotrichum gloeosporioides* and *Fusarium acutatum* respectively.

### Phylogenetic analysis

**Figure 3:**
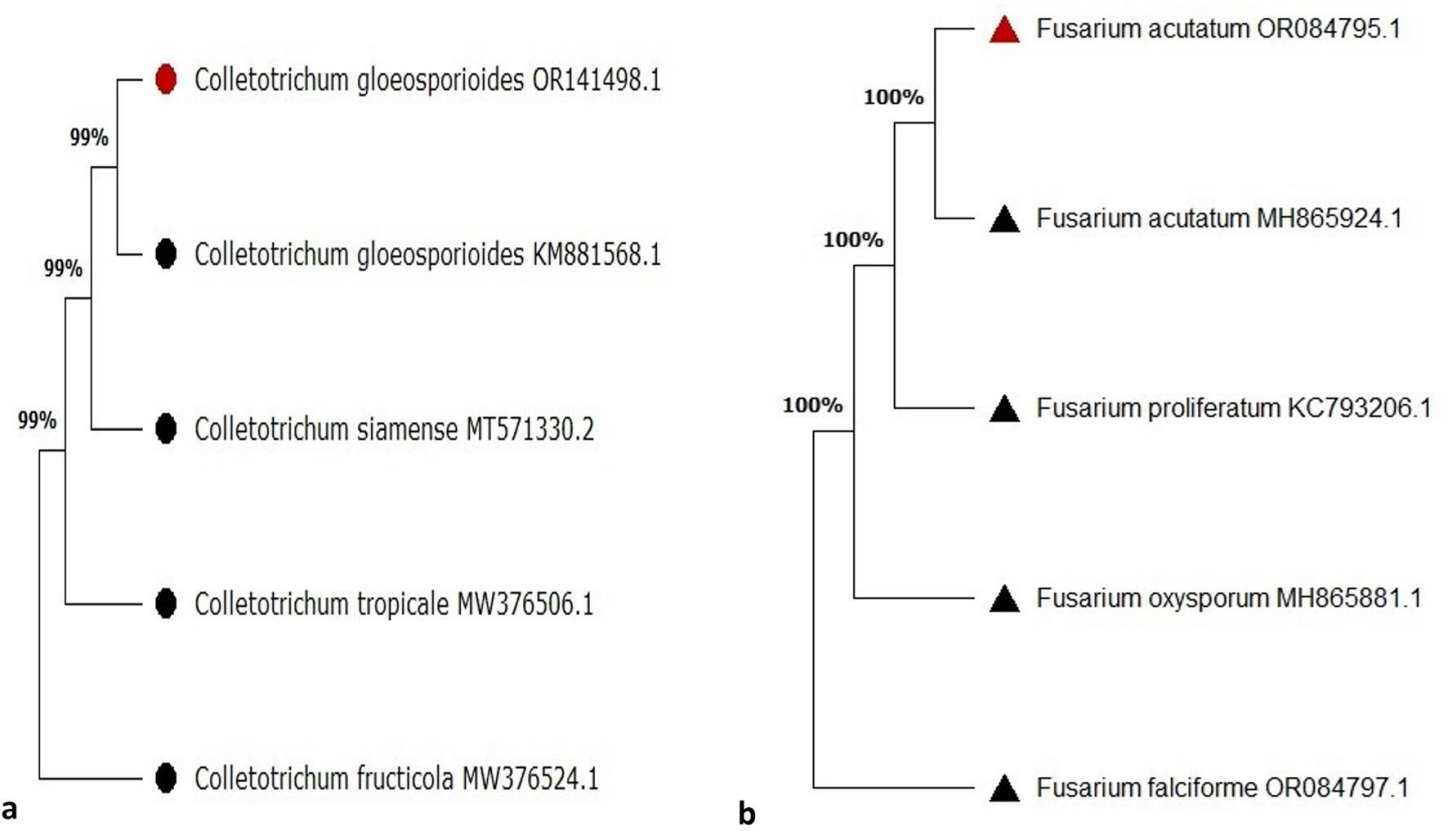
Phylogram using concatenated ITS and *tef* gene sequences of (**a**) *Colletotrichum* spp*.* and (**b**) *Fusarium* spp**.** The symbol of the sphere  in the figure indicates the *Colletotrichum* spp. and the symbol of triangle  indicates *Fusarium* spp. Red colored sphere  and triangle  indicate the isolates used in the present study.

To the best of our knowledge gene sequences of *C. gloeosporioides* and *F. acutatum* infecting the onion for twister disease are not available in the GenBank. The accession numbers of ITS and *tef* gene of *Colletotrichum gloeosporioides* (OR141498 and PP263370) and *Fusarium acutatum* (OR084795 and OR102876), respectively, (Fig. 3a and b) were used in phylogenetic analysis. The phylogenetic analysis based on multigene concatenated sequences clearly differentiated the species of *Colletotrichum* and *Fusarium* (Fig. 3). ITS and *tef* gene nucleotide sequences revealed that *C. gloeosporioides* and *F. acutatum* clustered in a distinct phylogenetic clade from other species of the *Colletotrichum* and *Fusarium*.

### Pathogenicity test

Artificial inoculations of pathogen/s and GA3 to onion seedlings were carried out as explained in the methods section. Symptoms developed 2 days after inoculation and were with 100% disease incidence in all inoculated plants and the results were given in Table 1.

**Table 1:**
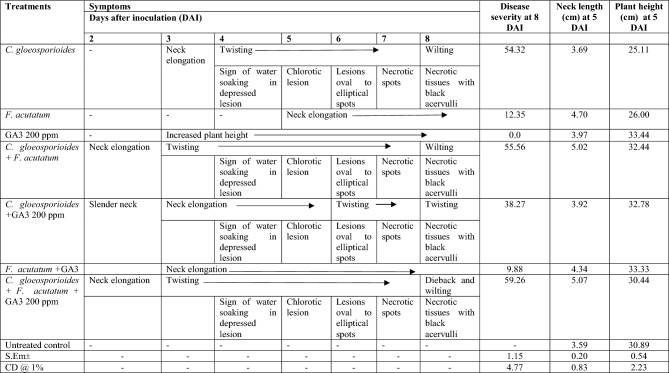
Development of infection of *C. gloeosporioides* and *F. acutatum* on onion plants

### *Colletotrichum gloeosporioides* alone

**Figure 4:**
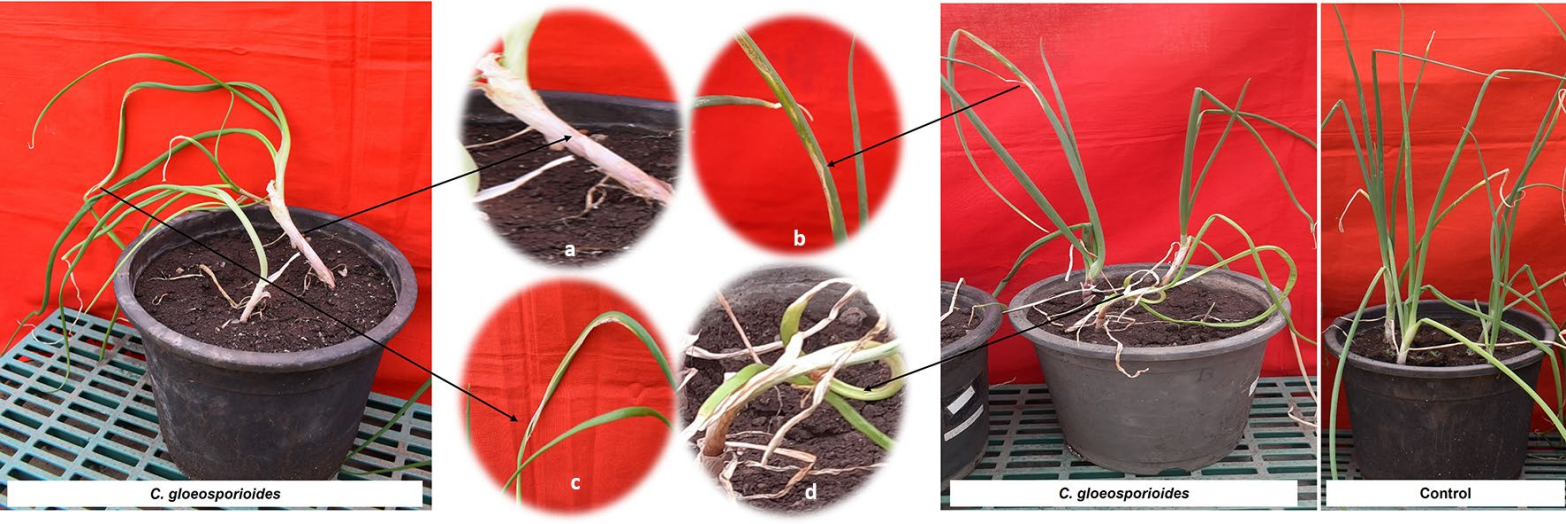
Pathogenicity assay of C. *gloeosporioides.* (**a**) Neck elongation. (**b**) Elliptical depressed spot. (**c**) Necrotic spots with black fruiting bodies. (**d**) Twisting**.**

The first sign, a slight neck elongation appeared three days after inoculation (DAI) while twisting of the leaves as well as the development of a water-soaking sign with depressed lesions on the leaves was observed on 4 DAI. Gradual elongation of lesions occurred from 5 DAI onwards and the leaves showed sunken oval lesions on the leaf blades while depressed lesions were observed on the culms. Later, these lesions developed into orange or salmon-colored conidial mass from 6 DAI, and on 7 DAI, these lesions became necrotic or matured. These lesions contained clusters of acervuli of *C. gloeosporioides* (8 DAI), which further led to the rotting of the culm. Wilting and dieback symptoms were also observed with a higher magnitude of disease (54.32%). At 5 DAI, the neck length was 3.69 cm which was not significantly different from control plants, but twisting of leaves was very evident in inoculated seedlings as compared to control (Fig. 4).

### *Fusarium acutatum* alone

**Figure 5:**
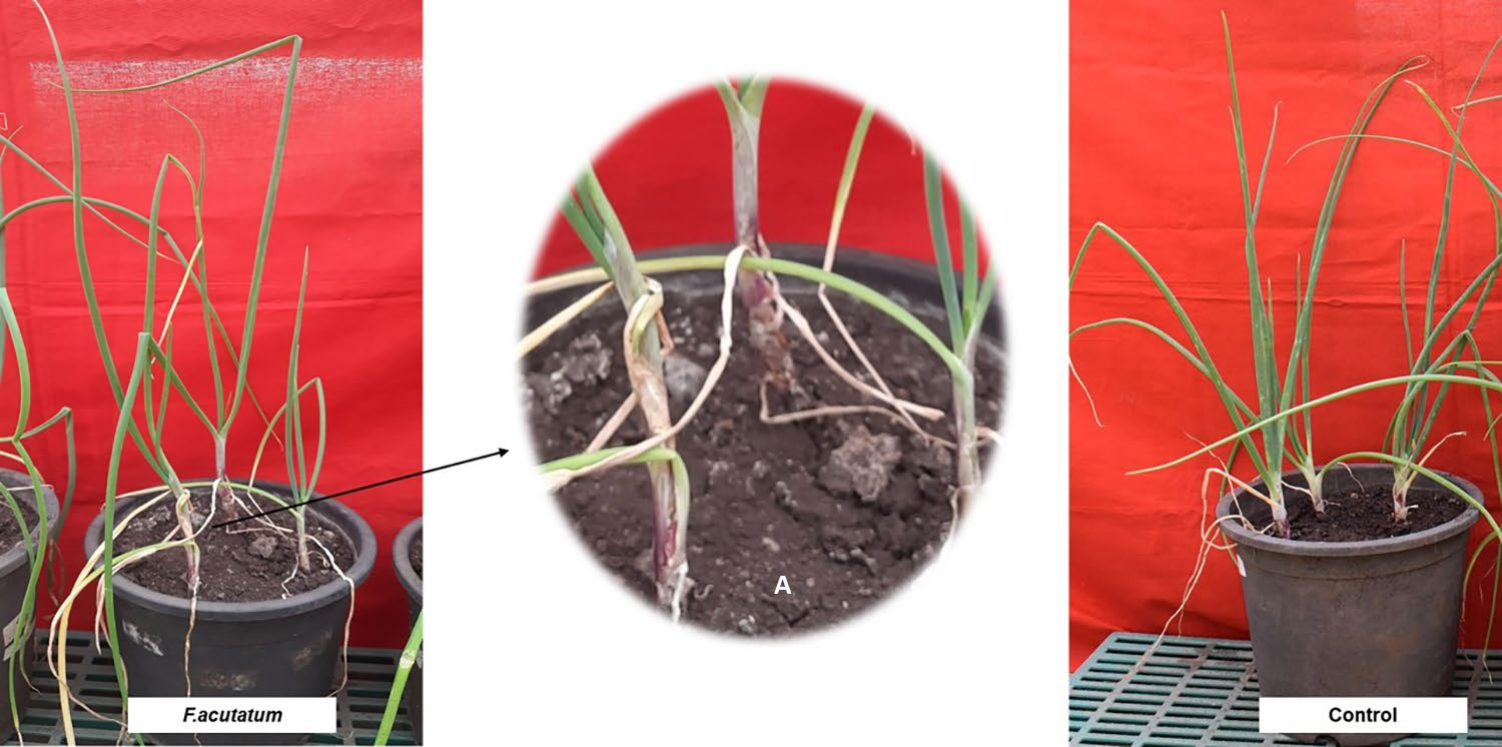
Pathogenicity assay of *F. acutatum* (**A**) Neck elongation.

There were no typical symptoms of twisting of leaves, however, all the inoculated plants induced neck elongation (4.7 cm) was observed from 5 DAI, plant exhibiting delay in infection against *F. acutatum.* Disease severity was correspondingly measured as 12.35% (Fig. 5).

### Gibberellic acid (GA3) alone

**Figure 6:**
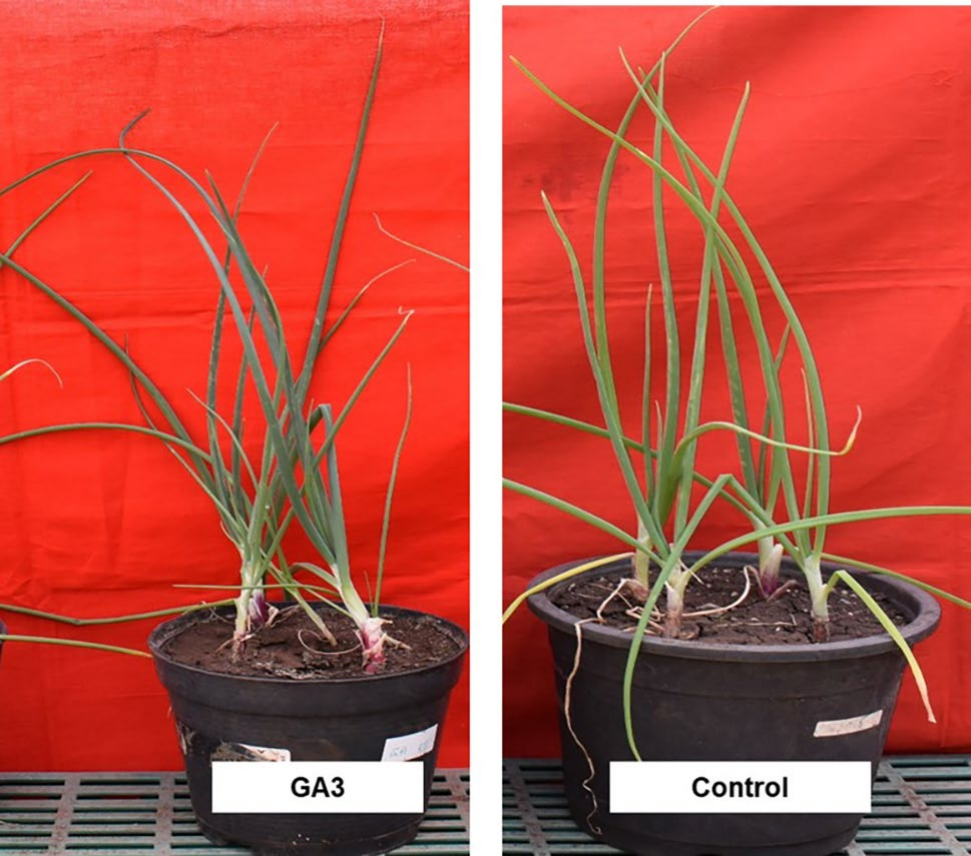
Pathogenicity assay of GA3 (Gibberellic acid), increased plant height.

Application of GA3 did not show any symptoms of twisting or lesion formation etc., however, the plant height (33.44 cm) and neck elongation (3.97 cm) were marginally increased in all the treated plants as compared to untreated control plants (Fig. 6).

### *Colletotrichum gloeosporioides* and *Fusarium acutatum* together

**Figure 7:**
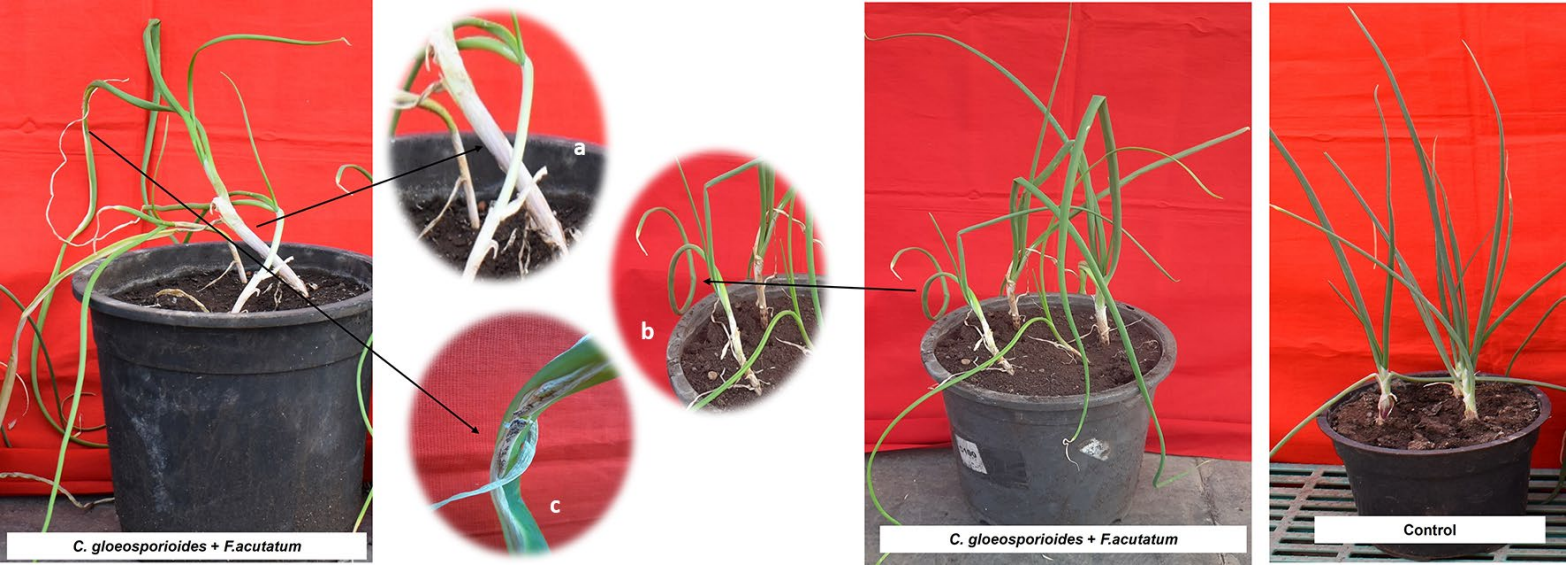
Pathogenicity assay of *C. gloeosporioides* + *F. acutatum,* (**a**) Neck elongation, (**b**) Twisting, (**c**) Necrotic spots with black fruiting bodies.

Combined inoculation of both *C. gloeosporioides* and *F. acutatum* induced neck elongation (2 DAI) and twisting (3 DAI) which were one day early as compared to *C*. *gloeosporioides* alone inoculated plants. Sign of water soaking in depressed lesions on leaves along with twisting was observed on 4 DAI. From 5 DAI these lesions became chlorotic and gradually elongated, sunken and oval at 6 DAI. Later, these lesions developed into orange or salmon colored conidial mass (7 DAI) and then these lesions became necrotic or matured containing clusters of black fruiting bodies (acervulli) of *C. gloeosporioides* (8 DAI), which led to wilting symptoms at final disease severity of 55.56% causing death of plants upon increasing severity. Early infection by combined application of pathogens as compared to independent inoculation has increased the magnitude of the disease (Fig. 7). The neck length and plant height were 5.02 cm and 32.44 cm, respectively at 5 DAI.

### *Colletotrichum gloeosporioides* and GA3 together

**Figure 8:**
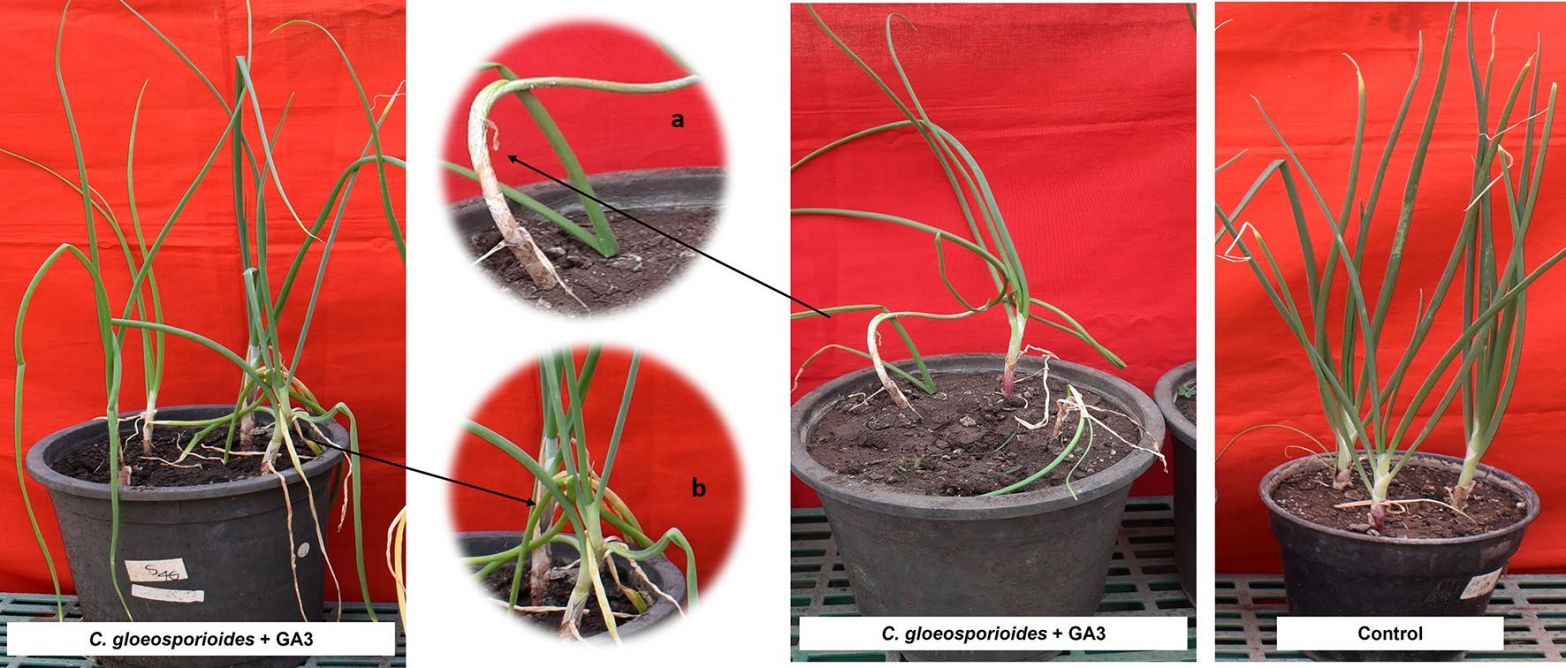
Pathogenicity assay of *C*. *gloeosporioides* + GA3. (**a**) Slender neck & neck elongation. (**b**) Twisting.

In the inoculated plants, only the sign of slender neck was observed on 2 DAI as compared to *C. gloeosporioides* alone inoculated plants. From 3 DAI the visible neck elongation was observed, and with neck elongation the sign of water soaking in depressed lesion was also observed from 4 DAI. Elongation of lesions occurred gradually by producing typical twister like symptoms from 6 DAI. The neck length of 5.02 cm with 32.44 cm plant height and twisting of leaves were evident from 5 DAI. From 6 DAI chlorotic lesions became sunken oval and developed with orange or salmon colored appearing conidial mass with twisting symptoms. From 7 DAI lesions became elongated and necrotic as matured. These lesions observed with clusters of acervulli of *C. gloeosporioides* (8 DAI) and the disease severity was reduced to 38.87% as compared to *C. gloeosporioides* alone inoculated plants. It is evident that the application of GA3 delayed the twisting symptoms for two days as compared to *C. gloeosporioides* alone inoculated plants where twisting symptoms observed on 4 DAI. The wilting was not observed in this combination of inoculation until 8 DAI of observation (Fig. 8).

### *Fusarium acutatum* and GA3 together

**Figure 9:**
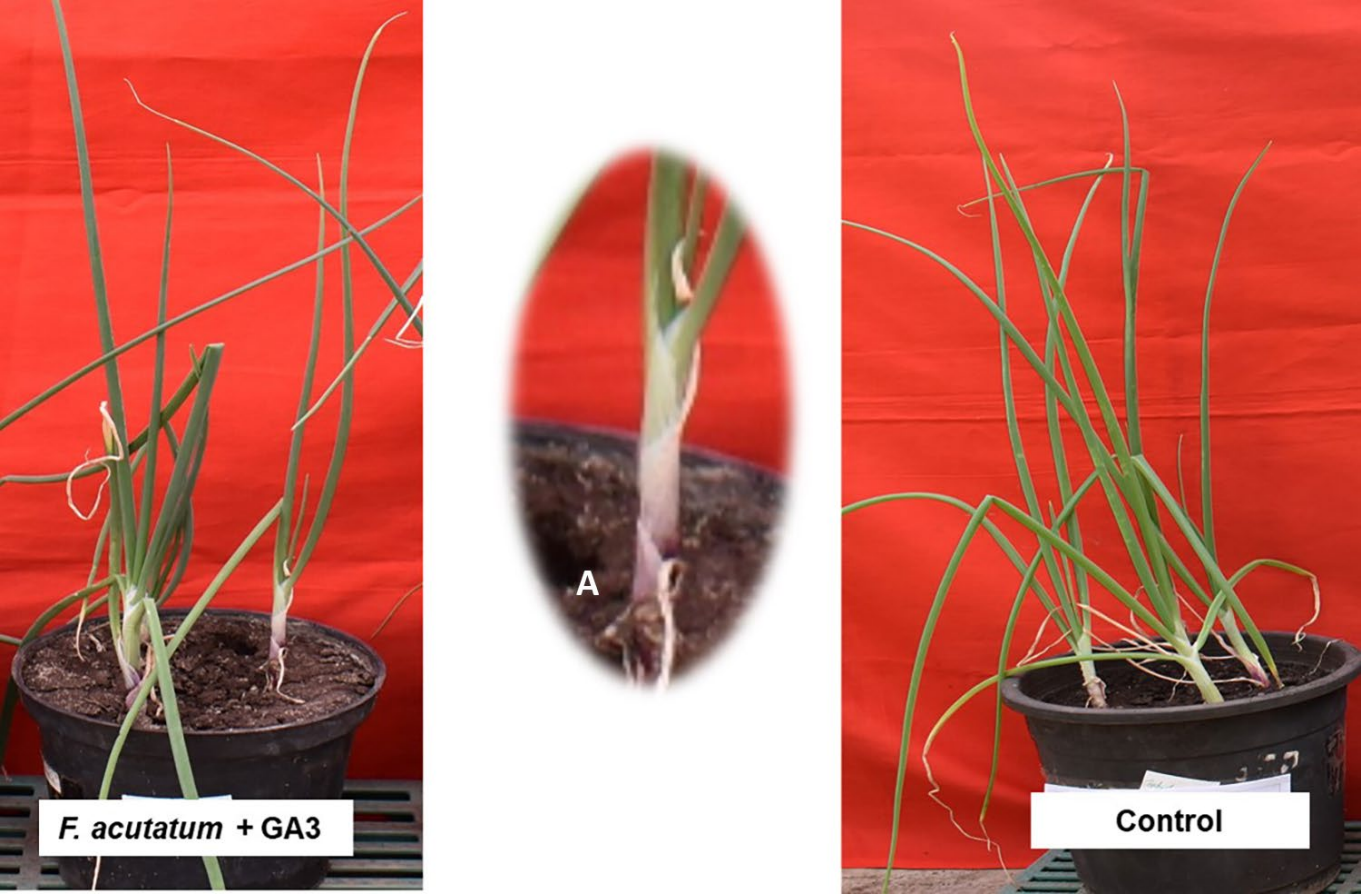
Pathogenicity assay of *F. acutatum* + GA3. (**A**) Neck elongation.

All plants were free from any kind of anthracnose-twister disease symptoms. However, there was slight neck elongation on 3 DAI, which might be due to GA3 influence on the neck elongation. The neck length and plant height were 4.34 cm and 33.33 cm, respectively on 5 DAI. The disease severity was reduced to 9.88% as compared to *Fusarium acutatum* alone inoculated plants (Fig. 9).

### *Colletotrichum gloeosporioides* and *Fusarium acutatum* added with GA3 altogether

**Figure 10:**
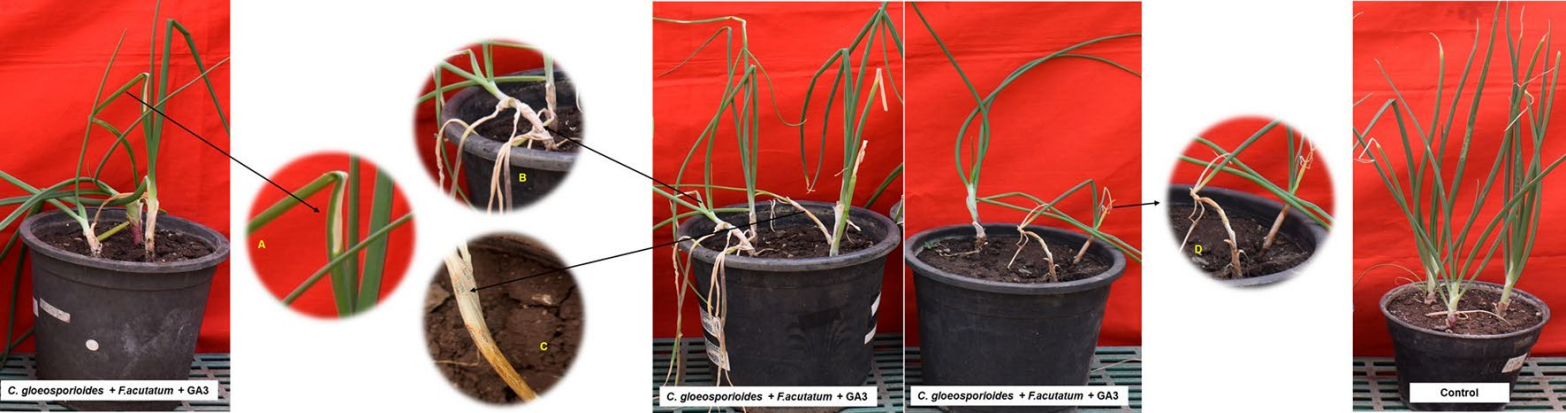
Pathogenicity assay of *C. gloeosporioides* + *F. acutatum* + GA3. (**A**) Elliptical depressed spot. (**B**) Neck elongation. (**C**) Necrotic spots with black fruiting bodies. (**D**) Twisting and dieback.

The pathogens and GA3 inoculated plants showed 100% disease incidence with disease severity of 59.26%. The inoculated plants exhibited the symptoms of neck elongation (2 DAI) and twisting (3 DAI) one day early as compared to C. *gloeosporioides* alone inoculated plants. The water soaking sign of depressed lesions appeared on leaves from 4 DAI. The lesions became chlorotic and elongation of lesions occurred gradually from 5 DAI. The leaves showed sunken oval lesions on the leaf blades and at the neck region. Later, these lesions developed into salmon/orange-colored conidial mass from 6 DAI, and on 7 DAI lesions became necrotic or matured and contained clusters of acervulli of *C. gloeosporioides* (8 DAI). At 5 DAI, numerically longest neck length (5.07 cm) was observed. However, the plant height (30.44 cm) recorded was a little lower as compared to *C. gloeosporioides* and *F. acutatum* combination (Fig. 10).

### Untreated control

Untreated plants sprayed with sterile distilled water did not receive infection and were free from any kind of the anthracnose-twister disease symptoms. The plant height of 30.89 cm and neck length of 3.59 cm was recorded on 5 DAI.

These results were revalidated and confirmed during 2023 and given in supplementary Table S1. Similar trend of the results was observed, however the duration of infection increased which may be due to time schedule of experiment where temperature change was visible during the experiment.

## Discussion

Anthracnose- twister is currently the major disease causing curling, twisting of leaves, chlorosis, abnormal neck elongation and anthracnose symptoms which were suspected to be caused by *Colletotrichum* spp. and *Fusarium* spp.^15^*C*. *gloeosporioides* and *F. acutatum* were isolated from onion leaves infected with anthracnose-twister disease following the procedures laid by Murtado et al.^22^ and Patil et al.^19^ The symptoms were slight curling and twisting of the leaves along with white depressed spots/lesions. It was observed that as the infection progressed, the lesions turned brown containing an orange mass of acervulli. Upon increase of severity, the disease complex further aggravated which ultimately led to the drying of leaves from tips giving wilting and dieback symptoms leading to bulb rotting and death of whole plant^15,17^. However, Alberto and Perez^23^, explained the symptoms of *C. gloeosporioides* infection in the field, starting with the appearance of a whitish lesion with an orange dot-like mass in the middle, the orange mass would then form concentric rings.

In our study the colony of *C. gloeosporioides* originally produced white to grey mycelia that darkened with age. Reecha et al.^14^ isolated *C. gloeosporioides* from shallot plants and also explained similar results where colonies were initially gray, which later became dark brown. The fungus also produced abundant conidia with cylindrical/dumbbell shapes as observed by Subramanyam^20^. The colony of *F. acutatum* produced white aerial mycelium and the macroconidia were septate, slightly curved or with bent apical cells and the microconidia were ovoid, aseptate, and chlamydospores were round, intercalary, hyaline and single, which were in agreement with Bhat et al.^24^

For further confirmation of identification, the pathogens were subjected to molecular characterization where ITS1/4 and *tef* genes were employed^25^. The sequences were blast searched in NCBI database confirming the identity of pathogens. The sequences were submitted to GenBank which are available with accession numbers of *C. gloeosporioides* (OR141498 and PP263370) and *F. acutatum* (OR084795 and OR102876) respectively. The phylogenetic analysis based on multigene concatenated sequences clearly differentiated the species of *Colletotrichum* and *Fusarium* (Fig. 3). ITS and *tef* gene nucleotide sequences revealed that *C. gloeosporioides* and *F. acutatum* clustered in a distinct phylogenetic clade from other species of the *Colletotrichum* and *Fusarium*. ITS is the most informative gene marker to differentiate *C. gloeosporioides* and *F. acutatum* from all other *Colletotrichum* spp. and *Fusarium* spp. and the present findings are similar as described by Patil et al.^19^ and Lestiyani et al.^18^

The pathogenicity test of *C. gloeosporioides* and *F. acutaum* caused anthracnose-/twister disease in onion variety, Bhima Super was done in green net house, and combined inoculation of *C. gloeosporioides*, *F. acutatum* and GA3 had little higher disease (59.26%). As per Lestiyani et al.^18^, *F. acutatum* is also one of the causative agents of twister disease, which we did not observe except neck elongation in our study.

*C. gloeosporioides* alone inoculated plants caused leaf twisting and anthracnose symptoms on 4 DAI and wilting as well as dieback symptoms were also observed, which were in agreement with Ebenebe^8^; Kanlong et al.^26^; Alberto^17^; Patil et al.^19^; Reecha et al.^14^ All processes including pre and post-penetration, the biological process of *C. gloeosporioides* in onion and the appearance of anthracnose symptoms could be completed within 8 DAI in this study. However, previous research says *C. gloeosporioides* has a short life cycle completing symptoms within 120 h after inoculation (5DAI) destroying crops in a very short period^13,17^. Differential climatic conditions and varietal differences might have caused slight delay in completing symptoms in this study.

Independent inoculation of *F. acutatum* did not produce twisting symptoms, however only neck elongation was observed from 5 DAI. However, Lestiyani et al.^26^ recorded the leaf twisting symptoms by *F. acutatum, F. solani* and *F. oxysporum*. The abnormal neck elongation and leaf twisting in onions was due to the synthesis and accumulation of gibberellins upon secondary infection with *G. moniliformis*^27^. In our study, *F. acutatum* infection did not produce twisting symptoms, which might be due to differential accumulation of hormones including gibberellins which needs to be investigated.

Plant hormones play a vital role in host–pathogen interactions, whether symbiotic or pathogenic. In our study, application of GA3 had growth promoting activity (increased plant height). According to Alberto^17^ GA3 plays a very important role in the disease development as it mimics the symptoms of the twisting disease upon application of GA3 100 and 1000 µg/ml in the neck of the onion seedlings producing severe elongation and twisting^19^. However, the role of the hormone in production of twisting is unclear and *Fusarium* spp. does not produce the hormone required to cause a severe twisting^18^. While these contrasting statements, in our study the role of GA3 was not observed for neck elongation or twisting symptoms. However, plant height increase was recorded.

Combined inoculation of *C. gloeosporioides* and *F. acutaum* induced the advance appearance of neck elongation by one day and produced twisting, anthracnose, and wilting symptoms. The early infection might be due to synergistic effects of both the pathogens making complex niches for disease development which increased the magnitude of disease. According to Alberto^17^, the combination of *C. gloeosporioides* + *G. moniliformis* caused severe damage in Yellow Granex, Red Creole and shallots.

In the combined *C. gloeosporioides* and GA3 inoculated plants, only the sign of slender neck was observed on 2 DAI and twisting seen at 6 DAI and there were no wilting symptoms, as compared to *C. gloeosporioides* alone inoculated plants. GA3 effect may be one of the reasons for the delay in appearance of symptoms. Our results are in conformity with Ebenebe^8^ and Kanalong^28^, which showed that onion twister is caused by *C. gloeosporioides* and till date there are no such reports for combined application of GA3 or other phytohormones with *Colletotrichum* to cause twister.

Inoculation of *F. acutatum* and GA3 together did not cause anthracnose-twister disease symptoms. However, there was slight neck elongation on 3 DAI, which might be due to GA3 influence on the neck elongation. *F. acutatum* alone or in combination of GA3 produced only neck elongation and no other twisting symptoms. *F. solani* is known to produce GA3^29,30^ which may affect the twisting disease^18^. The essential role of GA3 in plant growth for elongation cell of the stem, roots, flower, and fruit^31–33^ is well established. GA3 could induce the Bakanae disease^34^ by *Giberella fujikuroi*^35,36^.

Plants inoculated with *C. gloeosporioides* and *F. acutatum* with GA3 showed 100% disease incidence and exhibited the symptoms of neck elongation, twisting, anthracnose and wilting and increased the magnitude of the disease. In our study, twisting of onion was observed either in *C. gloeosporioides* alone treated plants or in combination of *F. acutatum*, and there was no role of GA3 either in neck elongation or twisting. May be GA3 is not produced by *F. acutatum*, which need further investigation. Alberto^17^ and Patil et al.,^19^ observed that *C. gloeosporioides* along with *G. moniliformis* and *C. gloeosporioides* added with *F. oxysporum* caused severe damage of twisting in onion. In our study, combined application of pathogens with GA3 slightly increased disease severity as compared to *C. gloeosporioides* and *F. acutatum* combination which might be due to differential host–pathogen interaction causing synergistic effect in the presence of GA3, which need further investigation.

The pathogenicity assays were helpful in determining the various symptoms as well as time period for completing the life cycle of the pathogen. Our findings showed that twister disease was caused by *C. gloeosporioides* alone and in combination with *F. acutatum*. Since *F. acutatum* might not produce GA3 it exhibited only neck elongation and no twisting symptoms. Combined inoculation of pathogens increased the severity of the disease. Our findings also confirm that both the pathogens in presence of GA3, could easily develop anthracnose-twister of higher magnitude taking only 8 days to complete its cycle. The results were revalidated and confirmed. Some of the results of our study like plant height and neck length did not follow any trend which warrant more intensive research involving multi ‘Omic’ studies.

## Methods

### Sampling, isolation and identification of *Colletotrichum* spp. and *Fusarium* spp.

Anthracnose-twister disease-infected plant samples were collected from the onion field (ICAR-DOGR, located at 18.5035 N and 73.5305 E, with an elevation of 611 m mean sea level, having a temperature range of 5.5—42.0 ºC and annual mean rainfall of 669 mm) during *Kharif* 2022 and were subjected to pathogen isolation using standard protocols^19,22^ and studied the cultural, morphological and molecular characters of the pathogen. Infected tissues were thoroughly washed with water, cut into 5 mm pieces, surface disinfected with 1% NaOCl for 1 min, further washed with sterile water, and incubated on PDA plates at 25 ± 2 °C for 7 days. Single hyphal tips were transferred onto fresh PDA plates in such a way as to obtain single-spore isolates. 5 mm mycelial plugs taken from three days old actively growing mycelia on PDA plates and slants were stored at 4 °C for further studies. The cultural and morphological characters were examined on 7-day-old culture using a compound microscope (Carl Zeiss).

### Molecular characterization of *Colletotrichum* and *Fusarium* sp.

*DNA extraction Colletotrichum* and *Fusarium* spp. were grown in potato dextrose broth for mycelium production to be used for DNA extraction. DNA was extracted from mycelium harvested from broth media based on cetyltrimethyl ammonium bromide (CTAB) method^37^ with slight modification. About 0.2 g of mycelium of each isolate was ground separately in a sterile pre-chilled mortar and pestle using liquid nitrogen to a fine frozen powder after which DNA extraction buffer (100 mM Tris HCl at pH 8.0, 1.4 M NaCl, 50 mM EDTA at pH 8.0 and 2% CTAB) was added. RNA present with DNA was removed by RNase treatment. Quantification of DNA was done by nano spectrophotometer. Working solutions having 25 ng/µl were prepared for optimization of polymerase chain reaction (PCR) by adding double sterilized nuclease free water.

### Polymerase chain reaction

The internal transcribed spacer (ITS1/4) and translation elongation factor (*tef*) alpha fragments were amplified using the primers pairs (5'-TCCGTAGGTGAACCTGCGG-3' and 5'-TCCTCCGCTTATTGATATGC-3') and (5'-CATCGAGAAGTTCGAGAAGG-3' and 5'-TACTTGAAGGAACCCTTACC-3'), respectively. The PCR reactions were carried out in 20 μl reaction mixture containing 10 × PCR buffer, 50 ng DNA template, 1.5 mM MgCl2, 0.25 mM dNTP mixture and 0.25 μM each of primer, and one unit of Dream Taq Polymerase (Thermo Scientific, India). PCR reactions were run in Applied Biosystems Thermocycler, USA with the following settings: Denaturation at 94 °C (5 min); then 35 cycles of 94 °C (30 s), 56 °C (45 s) and 72 °C (90 s). The final extension was done at 72 °C for 10 min.

### Sequencing and phylogenetic analysis

The ITS sequences so obtained were BLAST searched against the NCBI GenBank database to confirm the identity of the strains. The phylogenetic tree was established from concatenated ITS and *tef* gene sequences using the neighbor-joining (NJ) method in molecular evolutionary genetics analysis tool, MEGA ver. 11^38^. The branches with more than 50% confidence interval were selected, and the existing sequences of *Colletotrichum* (*C. gloeosporioides, C. fructicola, C. siamense* and *C. tropicale*) and *Fusarium* (*F. acutatum, F. falciforme, F. oxysporum* and *F. proliferatum*) available in the NCBI database were used to estimate similarity percentages. The sequences were deposited in the GenBank.

### Pathogenicity assay in controlled conditions

The pathogens, *Colletotrichum gloeosporioides* and *Fusarium acutatum* isolated from the host plant, cultured, mass-produced, and tested for their pathogenicity to the onion cultivar Bhima Super from where they were isolated. Before inoculation, the spore density of 7-day-old culture of the two pathogens was standardized (1 × 10^6^ spores/ml) and used for mass production. The seeds were thoroughly surface sterilized with 1% sodium hypochlorite for two minutes and washed in sterile distilled water, air dried, and sown in trays containing sterilized soil. Forty days old seedlings were used for the pathogenicity assay and the experiment was conducted in controlled conditions under a net house. In this assay, plants were inoculated with *C. gloeosporioides* or *F. acutatum* alone, combination of *C. gloeosporioides* and *F. acutatum,* and both pathogens along with GA3. The suspension containing *C. gloeosporioides* spores (1 × 10^6^ spores/ml) was sprayed on the entire plant and a giant culture of *F. acutatum* (10 g/kg soil) was applied to the soil. GA3 (200 ppm) was injected into the neck region of test plants using a sterile syringe according to Alberto^17^. The experimental design was entirely randomized and constituted in three replications. The control plants were treated with only sterile distilled water. Observations for pathogenicity starting from 2^nd^ day of inoculation until 8^th^ day were recorded daily. The appearance of typical twister and anthracnose symptoms on the leaves were recorded and then the fungi were re-isolated from the infected plants and cultured to satisfy Koch’s Postulates by checking their characteristics and sequence identity. Same set of experiment was conducted during 2023 by using same methodology. Disease reaction on the leaves was estimated on a grading scale of 1–9^15^ till eight days after inoculation.

### Statistical analysis

All the data from the experiment were statistically analyzed using WASP Stat. Statistical significance among different treatments was determined by one way ANOVA (Analysis of variance) followed by DMRT (Duncan’s Multiple Range Test) for statistical testing of comparison of means at *p*-value of 0.01.

### Research involving plants

All methods were carried out in accordance with relevant guidelines in the method section.

### Supplementary Information

Supplementary Tables.

## Data Availability

All data generated or analyzed during this study are included in this published article.
