# Supplementary material for: Insights into the cumulative effect of Colletotrichum gloeosporioides and Fusarium acutatum causing anthracnose-twister disease complex of onion
Source: Sci Rep. 2024 Apr 23;14:9374. doi: 10.1038/s41598-024-59822-w (PMC11039473; doi:10.1038/s41598-024-59822-w)
Supplement: Supplementary file 1 — Supplementary Tables. [file 41598_2024_59822_MOESM1_ESM.pdf]

Insights into the cumulative effect of C*olletotrichum gloeosporioides* and *Fusarium acutatum* causing anthracnose-twister disease complex of Onion.

Ram Dutta[1], Jayalakshmi K[1]*, Satish Kumar[1]*, Radhakrishna A[1]*, Manjunathagowda D. C[2], Sharath M. N[1], Vishal S. Gurav[1] and Vijay Mahajan[1]

[1] ICAR-Directorate of Onion and Garlic Research, Pune, Maharashtra, India

[2] ICAR-Indian Institute of Horticultural Research, Hesaraghatta Lake Post, Bengaluru, Karnataka, India[1]

***Corresponding author: jayalakshmipat@gmail.com

Table 1. Development of infection of *C. gloeosporioides* and *F. acutatum* on onion plants during *kharif* 2023

Treatments	Symptoms: Days after inoculation (DAI) 2	3	4	5	6	7	8	9	10	11	12	13	14	15	Disease severity (%) at 15 DAI	Neck length (cm) at 5 DAI	Plant height (cm) at 5 DAI
C. gloeosporioides	-	Neck elongation	Twisting →							Dieback		Wilting			55.56	2.87	31.37
			Sign of water soaking in depressed lesion	Chlorotic lesion	Elongation of spot turns to grey	Necrotic spots				Necrotic tissues with black acervulli							
F.acutatum	-	-	-	Neck elongation →											16.67	3.03	34.35
GA3 200 ppm	Increased plant height →														0.0	2.58	38.82
C. gloeosporioides + F. acutatum	-	Neck elongation	Twisting →						Dieback				Wilting		59.26	3.43	35.03
		-	Sign of water soaking in depressed lesion	Chlorotic lesion	Elongation of spot turns to grey	Necrotic spots	Necrotic tissues with black acervulli										
C. gloeosporioides +GA3 200 ppm	-	Neck elongation →			Twisting →			Dieback			Wilting				42.59	2.82	33.78
		Sign of water soaking in depressed lesion	Chlorotic lesion	Chlorotic lesion	Elongation of spot turns to grey	Necrotic spots	Necrotic tissues with black acervulli										
F. acutatum +GA3	-	Neck elongation →													11.11	3.10	35.40
C. gloeosporioides + F. acutatum + GA3 200 ppm	-	Neck elongation		Twisting →					Dieback		Wilting				64.81	4.35	34.40
	-	-	-	Sign of water soaking in depressed lesion	Cholorotic lesions	Lesions oval to elliptical spots	Necrotic spots with fruiting bodies										
Untreated control	-	-	-	-	-	-	-	-	-	-	-	-	-	-	-	2.47	31.88

Table 2. Weather factors during experiment

Days after inoculation	Temp Min (ºC)		Temp Max (ºC)		RH 1 (morning) (%)		RH2 (evening) (%)		Rain (mm)	
	Nov 2022	Sep 2023	Nov 2022	Sep 2023	Nov 2022	Sep 2023	Nov 2022	Sep 2023	Nov 2022	Sep 2023
2	15.20	22.70	30.20	30.20	72.00	87.00	53.00	69.00	0.00	0.00
3	14.70	21.70	29.20	28.70	62.00	87.00	52.00	76.00	0.00	0.00
4	13.20	22.20	31.20	30.20	71.00	90.00	52.00	80.00	0.00	2.00
5	15.20	22.20	29.70	29.20	77.00	90.00	54.00	72.00	0.00	23.00
6	18.20	22.20	31.70	29.20	81.00	84.00	63.00	72.00	0.00	5.00
7	15.20	22.70	31.20	30.20	81.00	82.00	62.00	78.00	0.00	0.00
8	14.20	21.20	30.70	29.20	69.00	83.00	63.00	81.00	0.00	0.00
9	14.20	19.70	29.70	30.70	81.00	80.00	64.00	74.00	0.00	0.00
10	13.20	21.20	30.20	29.20	82.00	83.00	66.00	71.00	0.00	0.00
11	15.20	20.20	30.70	29.70	73.00	83.00	65.00	68.00	0.00	0.00
12	16.70	19.70	32.20	30.20	91.00	80.00	62.00	83.00	0.00	0.00
13	14.70	21.70	31.20	28.20	85.00	87.00	66.00	80.00	0.00	0.00
14	14.20	20.20	30.70	26.70	76.00	95.00	56.00	80.00	0.00	5.80
15	13.20	27.20	29.20	24.20	62.00	87.00	57.00	76.00	0.00	1.80

Table 3. GenBank accession numbers of the different *Colletotrichum* and *Fusarium* isolates

Identity of isolates	GenBank accession used in phylogenetic analysis
Colletotrichum gloeosporioides	OR141498 (used in current study)
Colletotrichum gloeosporioides	KM881568
Colletotrichum siamense	MT571330
Colletotrichum tropicale	MW376506
Colletotrichum fructicola	MW376524
Fusarium acutatum	OR084795 (used in current study)
Fusarium acutatum	MH865924
Fusarium proliferatum	KC793260
Fusarium oxysporum	MH865221
Fusarium falciforme	OR084797
